# Cyber-Personality and *Liking* Expression: A Study From WeChat Users in China

**DOI:** 10.3389/fpsyg.2021.626040

**Published:** 2021-07-08

**Authors:** Haojian Li, X.T. Wang

**Affiliations:** School of Humanities and Social Science, The Chinese University of Hong Kong, Shenzhen, China

**Keywords:** cyberpersonality, communication motives, social media, WeChat, *liking* expression, personality traits

## Abstract

Clicking the *like* button following a post on social media has become a common means of expressing and gathering social support online. Little is known about how *liking* expression is linked and regulated by personality traits and communication motives. Following a preliminary survey (*n* = 168) about the usage of the *like* function on WeChat, a Chinese social media platform, we conducted an online study (*n* = 183) to map the Big-Five personality traits and five communication motives to the frequency (likelihood) of *liking* expression. The results showed that each user had, on average, 385 WeChat friends and spent 2.2 hours and used the *liking* function 1.1 times each day on WeChat. The personality trait *conscientiousness* was negatively related to the *liking* expression (β = −0.505, *p* < 0.05). In contrast, *agreeableness* promoted the expression of *liking* directly (β = 0.153, *p* < 0.05) and indirectly via two communication motives, *enjoyment* (a: β = 0.377, *p* < 0.01; b: β = 0.433, *p* < 0.001) and *passing time* (c: β = 0.578, *p* < 0.05; d: β = 0.523, *p* < 0.001). The *liking* expression may serve as a simple index for understanding dispositional underpinnings of social media networking.

## Introduction

### Cyber-Personality and Motives of Online Communication

Cyber-personality, human personality in cyberspace, has emerged as a new topic in social and personality psychology. The researchers have suggested that people's online behaviors, including online communication and online self-expression, are regulated by some specific personality traits and motivational factors (e.g., Anolli et al., 2005; Orchard and Fullwood, 2010; Attrill, 2015). In contrast to the vast amount of research on behavioral effects of personality in real life, little is known about how different personality traits manifest in the cyber-environment. The manifestations of personality traits and social motives in the cyber-environment may or may not be consistent with their behavioral effects observed in the actual world. The knowledge about personality effects in actual environments cannot be simply applied to cyber environments. For instance, a timid person in the real world may be highly aggressive in the cyber world. As the first step of the investigation, we intend to focus on a common and measurable online behavior and map it to specific personality traits as traditionally measured by the Big-Five personality inventory. In addition, we also map the online behavior to motivation constructs that drive and regulate social networking.

The online behavior examined in this research is the action of clicking the *like* button following a WeChat post, including, visual, auditory, or text message. WeChat is the most popular social media platform in China with over 1.2 billion monthly active users worldwide as of the first quarter of 2020, according to Statista.com. WeChat plays a dominant role in China's social media and influences people's online behavior, it enhances trust and personal contacts while transforming opportunities for larger online group formation (Harwit, 2017). WeChat users join friend circles, called “moments” which is similar to a Twitter post. The *like* function is one of the most common tools of expressing and gathering social support on WeChat. For this study, we chose this behavior because it is used readily by all the WeChat users to express social support. Secondly, the *like* clicking behavior is equally tractable and quantifiable for all its users.

In this research, we first establish a behavioral profile of *liking* behavior followed by a second study to analyze the relationship between the *liking* behavior and personality (i.e., the Big-Five personality traits) and motives underlying social networking (i.e., enjoyment seeking, pleasing others, and interpersonal relationship formation). Thus, the *like* expression may be used as a fast and frugal index of unique personality and social motive profiles. In addition, we examined which factors, *dispositional* or *situational*, are more significant regulators of the *like* expression behavior in social media.

### The *Like* Function in Communication on Social Media

The *like* function of WeChat provides a ready means for the users to selectively show individualized social support at their fingertips. Compared to *comment* on a post, which is a cognitively triggered behavior, *like* expression is more affectively driven (Kim and Yang, 2017). In general, the use of the *like* button reflects users' enjoyment of, agreement with, or interest in a specific post (Basalingappa et al., 2016). People within a social media group are not only *liking* others but also care about receiving *likes* from others. For the senders, *likes* signal social acceptance and maintain interpersonal relationships. For the receivers, *the likes* catch their attention with low cognitive load and have become a new source of social reward (Scissors et al., 2016). For adolescents, *likes* are considered a sign of social approval (Martinez-Pecino and Garcia-Gavilán, 2019). In particular, adolescents with little social support show a significant preference for these kinds of online social interactions (Leung, 2011). In general, *likes* serve as a cue of status and popularity (Blease, 2015). However, the overuse of social media may result in distress by increasing communication overload and reducing self-esteem (Chen and Lee, 2013).

Previous studies have primarily focused on the joint use of *liking, commenting*, and *sharing* and their communicational functions, based on data from Facebook, Twitter, or Instagram (Guo and Sun, 2020; Luo et al., 2020; Obamiro et al., 2020). The present study focuses on the link between the *like* expressions on WeChat and users' basic personality traits and communication motives. First, in a preliminary survey, we glean the behavioral profile of WeChat use in general and *liking* expression in particular. We then map personality traits and communication motives to the frequency of *like* expression.

### Personality Traits and Communication Motives and *Liking* Expression

To map the *like* behavior onto basic personality traits and communication motives of the WeChat users, we chose the widely-accepted Big-Five personality traits (Goldberg, 1992; John and Srivastava, 1999) as our personality measures. In user-generated online use of social media, users' personality traits play a crucial role in their engagement in and selection of social media.

Extraversion and openness to experience have been found to increase online activities and engagement in social media (Ross et al., 2009; Correa et al., 2010). However, other studies found that some extroverts may use social media for social enhancement and some introverts may use social media to compensate for the lack of social interactions in face-to-face contexts (Zywica and Danowski, 2008). For neuroticism, some researchers believe that a high level of neuroticism had a greater impact on messaging use and online texting. Neurotic individuals reported stronger addictive tendencies in mobile phone use (Ehrenberg et al., 2008). However, other studies showed that neuroticism was unrelated to online activities and engagement in social media (Ross et al., 2009). For agreeableness, it was reported that a low score was associated with low involvement in social media (Landers and Lounsbury, 2006) and a higher intention for maintaining existing relationships (Horzum, 2016).

Regarding motivations of the consumers of social media, the literature suggests two major motives, enjoyment seeking and interpersonal relationship maintenance, derived from an influential theory in the field of communication, *uses and gratifications theory* (Rubin, 1994; Ruggiero, 2000; Quan-Haase and Young, 2010; Whiting and Williams, 2013). The theory explains how people use social media to gratify their needs. According to the theory, users select different media based on their motivational needs. In a more recent study, Lee et al. (2016) identified from the literature five types of motives that make the users click the *like* button on Facebook. Of the five communication motives (i.e., enjoyment, pleasing others, monetary incentive, pass time, and interpersonal relationship), enjoyment, and interpersonal relationship are the two most salient motives. A cross-cultural study with Chinese users on WeChat (Gan and Wang, 2015) also confirmed that the two major types of gratifications, besides finding content information, were hedonic and social gratifications. In the present study, we focused on the aforementioned five communication motives because they are unique for social media use and also overlap with basic human needs (Maslow, 1954).

Quan-Haase and Young (2010) reported that Facebook use is associated more with having fun and knowing about the social activities occurring in one's social network, whereas *instant messaging* is geared more toward relationship maintenance and development. For this study, we examine not only what type of need the *like* function on WeChat fulfill but also how motives and personality traits separately or jointly affect the users to click the *like* button on WeChat.

Our research questions derived from the aforementioned discussion are as follows:

RQ1. How do people use the *like* function on WeChat?
RQ2. Is there any particular personality trait that regulates the use of *like* expression?
RQ3. What communication motives are driving the use of the *like* function?

Although we do not have strong predictions regarding the aforementioned research questions, we hypothesize that the use of the *like* function on WeChat will be affected by the user's personality and communication movies. In particular, two of the Big-Five personality traits, agreeableness and conscientiousness would likely affect the use of *liking* expressions. While agreeableness may increase the use of the *like* function (e.g., Landers and Lounsbury, 2006), conscientiousness may reduce the use of the *like* function due to a more cautious and responsible attitude. In addition, we predict that the effects of agreeableness on the use of the *like* function would be mediated by communication motives, particularly, the enjoyment-seeking motive, as suggested by some previous findings (Lee et al., 2016).

## Materials and Methods

### Participants

In this research, two samples were collected from university students in Beijing. We conducted a preliminary survey to learn how the young participants use WeChat and the *like* function (*n* = 168, 50 male participants). For the main study, the sample consisted of 190 volunteer participants who use WeChat daily. Seven participants failed to complete the survey and were excluded from data analysis. The age of the main sample (*n* = 183) ranged from 17 to 23 years, with an average age of 18.3 ± 0.91 years. This research was endorsed by the IRB of the university and conducted before the outbreak of the COVID-19 pandemic.

### Measures

#### Preliminary Survey Questionnaire

The questions in the survey gathered basic information about the usage of WeChat and the *like* function, including the number of friends on WeChat, the time spent each day on WeChat, the frequency of using the *like* function, the percentages of the WeChat posts that the participants agree, trust, and considered useful, etc. (for more details see Appendix).

#### Big-Five Personality Scale

In the main study, we measured the Big-Five personality traits using the 60 items of the NEO-FFI inventory (Costa and McCrae, 1992). The inventory assesses five personality traits: extraversion, agreeableness, conscientiousness, emotional stability, and openness to experience. The Chinese version of this inventory had been used and tested previously (Yao and Liang, 2010). Participants responded to the items on a 5-point Likert scale (1 = strongly disagree to 7 = strongly agree). The Cronbach's alpha coefficients in the present study were 0.86 for extroversion, 0.83 for conscientiousness, 0.70 for agreeableness, 0.66 for openness, and 0.81 for neuroticism (emotional stability).

#### Communication Motives for Using the *Like* Function

We adopted a 21-item inventory developed by Lee et al. (2016) to measure how the *like* expression on WeChat is related to the following five communication motives: enjoyment, pleasing others, monetary incentive, passing time, and interpersonal relationship. The questionnaire begins with an incomplete statement “I click *like* on WeChat because …” followed with 21 items, such as “I enjoy the content,” “It helps me fit in with a group of people,” “It helps me receive a bargain deal,” “It helps me pass time when I am bored.”, and “It helps me improve relations with friends.” The participants rated these statements on a 7-point scale with 1 meaning strongly disagree and 7 meaning strongly agree. In this scale, the Cronbach's α is 0.79 for *enjoyment*, 0.87 for *pleasing others*, 0.93 for *money incentive*, 0.82 for passing time, 0.91 for *interpersonal relationship*.

#### Dependent Variable

The dependent variable was a frequency rating of *like* expression on WeChat. The participants were asked: “How frequently do you use the *like* function on WeChat?” The response was measured on a Likert-type scale with the following response anchors: 1 = never, 2 = rarely, 3 = occasionally, 4 = sometimes, 5 = often, 6 = usually, 7 = always (Vagias, 2006). We chose this Liker-type response instead of an absolute estimation such as the number of *like* clicking each day which is likely to vary from time to time and be subjective to memory biases. The current measure is a numerical likelihood estimate in comparison to other WeChat users.

### Procedure and Data Analysis

We first conducted a preliminary survey to obtain a WeChat user profile. In the following main study, the questionnaires were distributed online. The consent for participation was obtained before answering the questions. The personality and motivation measures were presented in a balanced order.

Descriptive statistics, Pearson's correlations with Benjamini-Hochberg correction, were calculated. We then conducted a hierarchical regression analysis to reveal the unique contributions of each set of factors (i.e., Big-Five personalities, communication motives) to the model, followed by a path-analysis to sort out the relationships between Big-Five personality traits, communication motives, and *liking* expression on WeChat.

## Results

### The Results From the Preliminary Survey

To answer RQ1, the preliminary survey gathered basic information to obtain a WeChat user profile. We also asked the participants for their basic attitude toward, perception of, and emotional experience with the *like* function on WeChat (see Appendix).

In general, the frequency of using *like* is much higher than using some other functions (e.g., retweet, comment, and create a new post). The frequency of *liking* behavior was positively correlated with other endorsement behaviors such as retweets. The use of the *like* function was considered a means of expressing immediate positive feedback.

Each user had, on average, 385.4 ± 322.5 friends on WeChat and spent 2.21 ± 1.19 hrs and used the *liking* function 1.1 ± 0.52 times each day on WeChat. Of the posts consumed by the participants, 62% were considered trustworthy, 57% were agreeable, and 44% were considered meaningful. The participants would use the *like* function only when they liked or strongly liked a post. The frequency rating exceeded the middle point on the response scale (see Appendix).

These results suggest that WeChat users did not abuse the *like* function which is easy to use at their fingertips. The rate of re-posting a message was even lower, which was about once every 3 days.

### Regression Analysis of Personality Traits, Motives, and the *Like* Expressions

Table 1 shows the means, standard deviations, and inter-correlations between the Big-Five personality traits, five communication motives, and the frequency rating of *like* expression on WeChat. To control the family-wide type-1 error, we conducted Benjamini-Hochberg correction to the predictors of *Like* expression (see *p*-values with Benjamini–Hochberg correction in Table 1). The frequency rating of *like* expression was positively correlated with the personality trait *agreeableness* and all five communication motives. and negatively correlated with the personality trait *conscientiousness* (not significant after Benjamini–Hochberg correction).

**Table 1:** Means, standard deviations, intercorrelations between sex, age, personality traits, communication motives, and liking expression, and *P*-values with Benjamini–Hochberg correction.

		**1**	**2**	**3**	**4**	**5**	**6**	**7**	**8**	**9**	**10**	**11**	**12**	**13**
1	Gender	1												
2	Age	0.04	1											
3	Openess	−0.08	−0.05	1										
4	Agreeableness	0.05	−0.06	−0.02	1									
5	Conscientiousness	−0.04	−0.10	0.02	0.30^**^	1								
6	Extroversion	0.03	−0.15^**^	−0.05	0.46^**^	0.47^**^	1							
7	Neuroticism	0.07	0.03	0.03	−0.41^**^	−0.38^**^	−0.56^**^	1						
8	Enjoyment	−0.19^**^	−0.08	0.00	0.20^**^	0.05	0.13	−0.01	1					
9	Pleasing others	0.03	−0.06	−0.09	0.16^*^	−0.15^*^	0.06	−0.10	0.31^**^	1				
10	Money incentive	0.06	−0.05	−0.19^**^	0.13	−0.02	0.02	−0.10	0.19^**^	0.49^**^	1			
11	Passing time	−0.11	0.01	−0.15^*^	0.16^*^	−0.1	0.08	0.02	0.15^*^	0.38^**^	0.21^**^	1		
12	Interpersonal Relation	−0.11	−0.10	−0.08	0.18^*^	−0.07	0.001	0.02	0.49^**^	0.64^**^	0.30^**^	0.38^**^	1	
13	*Liking* Expression	−0.14	−0.02	−0.08	0.21^**^	−0.15^*^	0.06	0.02	0.29^**^	0.34^**^	0.16^*^	0.54^**^	0.35^**^	1
	*p*-values^**§**^	ns	ns	ns	0.015	ns	ns	ns	0.00036	0.00001	ns	0.00001	0.00001	
Mean		1.72	18.84	3.38	3.397	3.43	3.3	2.98	5.30	3.25	3.18	3.57	4.43	4.02
SD		0.45	0.91	0.42	0.444	0.57	0.64	0.61	0.83	1.23	1.75	1.66	1.33	1.84

*N = 183*.

* *p < 0.05*;

** *p < 0.01*.

§ *p-values with Benjamini–Hochberg correction*.

*ns, not significant*.

To test our hypothesis regarding RQ2 and RQ3 and to further sort out the relative contributions of the personality and motivational factors on *like* expression, we conducted hierarchical regression analysis.

Table 2 shows the results of hierarchical regression analysis with the frequency rating of *liking* expression as the dependent variable. We conducted a hierarchical regression analysis to reveal the unique contributions of each set of factors (i.e., Big Five personalities, communication motives) to the model. We chose hierarchical regression to allow hypothesis-based placement of variables into the model instead of automated and algorithm-based analysis, such as stepwise regression. Gender and age were entered in the first step, followed by adding the personality traits at the second step, and the five communication motives in the third step. Communication motives were placed into the hierarchical regression following the personality measures based on the consideration that the motives of communication are more domain-specific than the Big-Five personality traits and are expected to mediate the effects of personalities.

**Table 2:** Hierarchical regression results of the contributions of age and gender (first step), personality traits (second step), and communication motives (third step) to *liking* expression on WeChat.

**Model**		**Unstandardized coefficients**	**Standardized coefficients**	**VIF**
		**β**	**β**	
**Liking expression on WeChat**				
1	Gender	−0.566	−0.139	
	Age	−0.026	−0.013	
2	Gender	−0.743	−0.183^*^	1.028
	Age	−0.023	−0.011	
	Neuroticism	0.359	0.12	
	Extroversion	0.3	0.105	
	Conscientiousness	−0.806	−0.250^**^	1.337
	Agreeableness	1.231	0.297^***^	1.341
	Openness	−0.384	−0.087	
3	Gender	−0.331	−0.081	
	Age	−0.011	−0.006	
	Neuroticism	0.133	0.045	
	Extroversion	0.041	0.014	
	Conscientiousness	−0.456	−0.142	
	Agreeableness	0.669	0.162^*^	1.454
	Openness	−0.078	−0.018	
	Pleasing others	0.156	0.104	
	Money incentive	−0.033	−0.032	
	Passing time	0.477	0.431^***^	1.319
	Interpersonal relation	0.001	0	
	Enjoyment	0.341	0.153^*^	1.456
	*R*^2^ step 1	0.02	Adjusted *R*^2^ step 1	0.09
	*R*^2^ step 2	0.142	Adjusted *R*^2^ step 2	0.107
	*R*^2^ step 3	0.378	Adjusted *R*^2^ step 3	0.334
	*R*^2^ total	0.54	Adjusted *R*^2^ total	0.531

*N = 183*.

* *p < 0.05*;

** *p < 0.01*;

*** *p < 0.001*.

Concerning the *liking* expression, age and gender only accounted for 2% of the variance in the dependent variable. No sex-personality trait interaction or sex-communication motive interaction effects were detected.

The predictability of the model improved significantly by adding the personality traits, which accounted for a further 14.2% of the variance. The motivation traits accounted for another 37.8% of the variance. The total percentage of the variance accounted for by the full model reached 54%. In Table 2, we reported *R*^2^ and Adjusted *R*^2^ for model fitting and tested multicollinearity using the variance inflation factor (VIF). The VIF scores (far right column) were all lower than 2, indicating that the predictors included in the multiple regression were not highly correlated with each other.

### Direct and Indirect Effects of Personality Traits and Communication Motives on the *Liking* Expression

To further test our hypothesis about mediation effects of motivation between personality and the use of the *like* function, we conducted path analyses using M-plus and revealed direct and indirect effects of personality and motivation on *liking* expressions.

**Figure 1:**
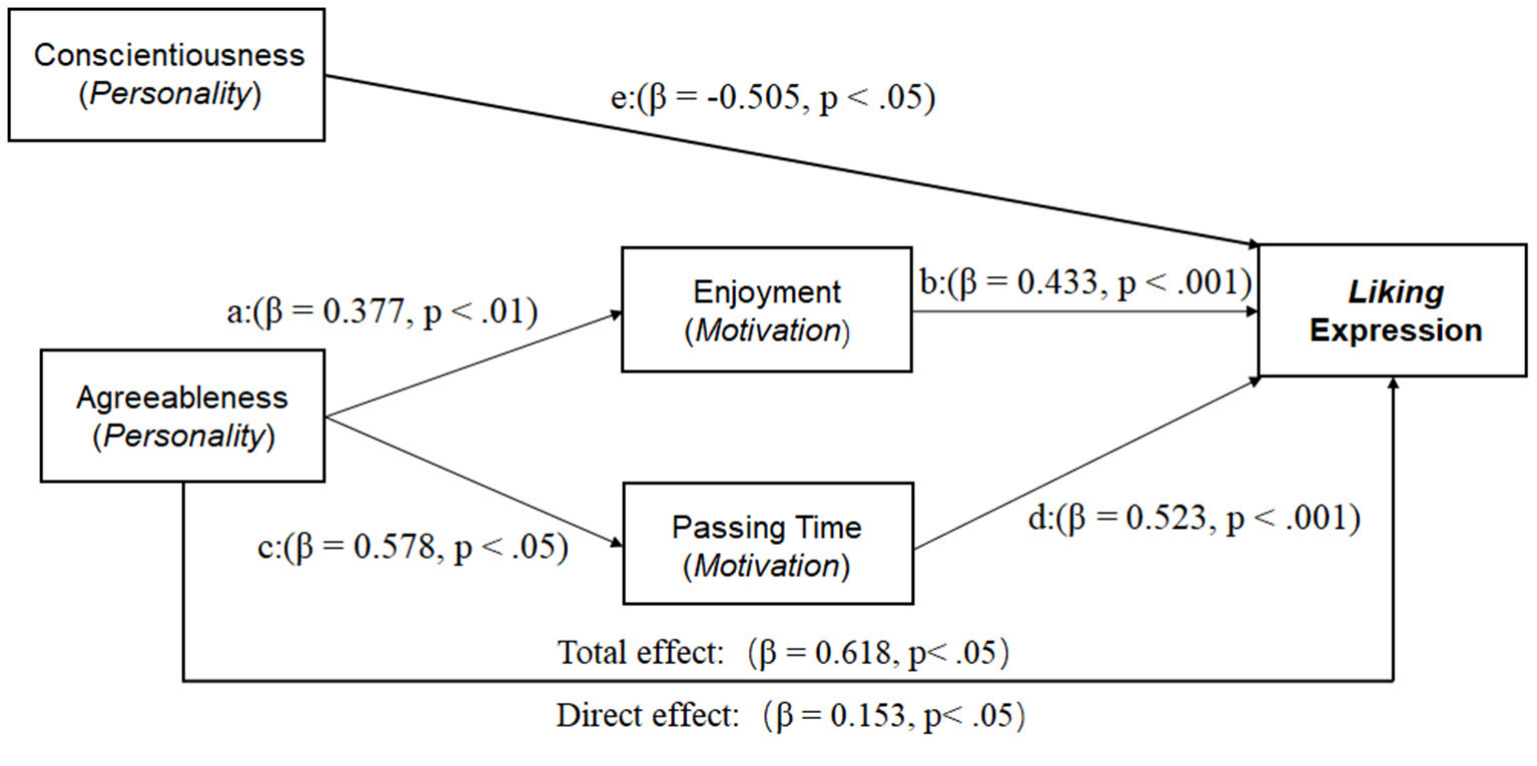
Relationship between personality, motivation, and *liking* expression.

As shown in Figure 1, the personality trait *conscientiousness* was negatively related to the *liking* expression (β = −0.505, *p* < 0.05), suggesting that higher conscientiousness makes a public expression of social support more serious and less casual. In contrast, another Big-Five personality trait *agreeableness* promoted the expression of *liking* directly (e: β = 0.153, *p* < 0.05) and indirectly via two communication motives, enjoyment, and passing time. First, the participants who had higher conscientiousness perceived themselves to be less likely to use the liking expression. This direct effect suggests that agreeableness lowers the threshold for a person to express social support in a cyber-environment.

Second, the positive effects of agreeableness on the *liking* expression were mediated by two communication motives. One of the mediating motives was the enjoyment motive, with a path from agreeableness to enjoyment motive (a: β = 0.377, *p* < 0.01) and then from the enjoyment motive to the *liking* expression (b: β = 0.433, *p* < 0.001). A bias-corrected bootstraps confidence interval for the indirect effect (ab = 0.163) based on 5,000 bootstrap samples was entirely above zero (0.012–0.315). This mediation effect suggests that using the *liking* function on social media was a pleasant experience and self-rewarding.

The second mediating factor was the motive of passing time, with a link from agreeableness to the motive (c: β = 0.578, *p* < 0.05) and a final link from the motive to the *liking* expression (d: β = 0.523, *p* < 0.001). This indirect pathway shows that the *liking* expression on social media provides a way of killing time. A bias-corrected bootstraps confidence interval for the indirect effect (cd = 0.302) based on 5,000 bootstrap samples was entirely above zero (0.012–0.592).

The effect of agreeableness on like expression was reduced after controlling the two motives but remained statically significant (see the outer path in Figure 1, *p* < 0.05). Therefore, the result indicates a partial mediation effect between agreeableness and the *liking* expression via the two communication motives. Moreover, the mediating effects of the two motives were especially stronger for a personality of higher agreeableness and lower conscientiousness. The whole model with two partial mediating pathways and one direct pathway had a significant fit of data (*R*^2^ = 0.337, *p* < 0.001).

## General Discussion

We derived from the data a four-factor model of dispositional effects on the *lik*ing expression in communication on social media (see Figure 1). Consistent with our hypothesis, the personality traits *agreeableness* and *conscientiousness* had, respectively, positive and negative effects on *like* expression. Moreover, the hypothesis that the communication motives mediate the effects of personality traits on the *liking* expression is partially supported where only a single trait (agreeableness) was mediated by leisure-related communication motives.

What the *liking* function is not about should be also important to learn. *Liking* expressions are neither associated with introversion-extraversion nor emotional stability nor openness to experience. In the present study, the sores of extraversion were not significantly correlated with the use of the *like* function on WeChat. The subjective frequency estimate of *like* expression did not distinguish introverts from extroverts. Both introverts and extroverts may benefit from using the *like* function for different reasons. Introverts may benefit from the *like* function to build up their social relations without face-to-face interactions while extroverts may use the *liking* expression for social enhancement (Zywica and Danowski, 2008).

The use of the *liking* function on social media is neither for simply pleasing others nor for utilitarian purposes. In a follow-up study, we asked a group of student participants about the time they spent and the number of the *like* expressions they used each day on WeChat during the COVID-19 pandemic. The results showed that both measures were not statistically different before and after this environmental change. Thus, the use of the *liking* function on social media was not easily susceptible to situational changes in one's environment, indicating it is more of an index of dispositional than situational factors.

Overall, our results indicate that the *liking expression* is more dispositional than situational. It is regulated by personality traits and communication motives rather than situational factors (e.g., environmental changes, health risks, and presumably emotional state due to the COVID-19 pandemic). The findings of the present study suggest that people did not abuse the easy function of *liking* expression on social media. Instead, expressing such social support is regulated by conscientiousness and agreeableness, and mediated by communication motives of passing time and enjoyment seeking.

### Limitations and Future Directions

This research has its limitations in its design. First, a clear limitation of this study is its limited demographic sample with little age variation. Our sample was homogeneous, including mainly young university students. These participants are likely to be more active in cyberspace, whose communication motives may be different from other user groups. Second, our measure of the use of the *like* function was a likelihood estimate in comparison to other users in one's friend circle.

People use the *like* function on WeChat for both light-hearted topics and more serious topics. It is plausible and can be examined in future studies that agreeableness is associated more with light-hearted topics while conscientiousness is associated more with serious topics.

Future studies should adopt multiple measures, including the actual frequency measure, attitude toward using the *like* function, and self-confidence measures of social media networking. A comprehensive measure of *liking* expressions may yield a more reliable and valid index for the cyber-personality profiling of the users of social media.

## Conclusion

The current study is the first to build the user profile of liking expressions on WeChat and to link the *liking* expression to its underlying personality traits and communication motives. The results showed that the *liking* expression is only linked with limited personality traits (conscientiousness and agreeableness) and communication motives (passing time and enjoyment seeking) and is more sensitive to these dispositional factors than situational factors, such as changes due to the COVID-19 pandemic. The unique user profile associated with the *liking* expression makes it a simple and readily available tool for predicting particular communication needs and personal characteristics of social media consumers. The frequency of *liking* expressions reveals the dispositional underpinnings of individual users and social media groups.

## Data Availability Statement

The raw data supporting the conclusions of this article will be made available by the authors, without undue reservation.

## Ethics Statement

The studies involving human participants were reviewed and approved by the Ethics Committee of the School of Humanities and Social Science, Chinese University of Hong Kong, Shenzhen. The participants provided their informed consent to participate in the study.

## Author Contributions

HL: methodology, investigation, data curation and analysis, and writing- original draft preparation. XW: conceptualization, methodology, validation, resources, data analysis, writing- reviewing and editing, project supervision, and funding acquisition. All authors contributed to the article and approved the submitted version.

## Conflict of Interest

The authors declare that the research was conducted in the absence of any commercial or financial relationships that could be construed as a potential conflict of interest.

## Appendix

**Table A1:** A user profile of Liking expressions on WeChat (*n* = 168) with descriptive statistics of frequency estimates, likelihood (1 = rare, 2 = unlikely, 3 = possible, 4 = likely, 5 = almost certain) and agreement (1 = agree and 5 = disagree) ratings on a 5-point scale.

	**Minimum**	**Maximum**	**Mean**	**SD**
1. How long in hours do you use WeChat each day?	1	5	2.2	1.2
2. How many WeChat friends do you have?	30	3,000	385.0	322.5
3. How many *like* expressions do you send to other's posts (e.g., text, video, image, article) every 3 days?	1	8	3.3	1.6
4.1. Out of every 10 posts you read on WeChat, how many of them do you agree with?	2.0	9.0	5.7	1.3
4.2. Out of every 10 posts you read on WeChat, how many of them do you think are meaningful?	1.0	9.0	4.4	1.7
4.3. Out of every 10 posts you read on WeChat, how many of them do you trust?	1.0	9.0	6.2	1.6
4.4. Out of every 10 posts you read on WeChat, how many of them do you feel passionate about?	1.0	8.0	4.5	1.6
5.1. How likely are you to click the *like* button to endorse a post you somewhat like?	1.0	5.0	2.9	0.9
5.2. How likely are you to click the like button to endorse a post you like?	1.0	5.0	3.3	0.9
5.3. How likely are you to click the like button to endorse a post you strongly like?	1.0	5.0	4.0	1.0
6.1. How likely are you to click the like button to endorse a post you somewhat dislike?	1.0	5.0	2.1	0.9
6.2. How likely are you to click the like button to endorse a post you dislike?	1.0	5.0	2.3	1.1
6.3. How likely are you to click the like button to endorse a post you strongly dislike?	1.0	5.0	2.8	1.3
7.1. When you agree with a post, how likely are you to click the *like* button?	1.0	5.0	4.1	0.8
7.2. When you agree with a post, how likely are you to make a comment?	1.0	5.0	3.3	1.0
7.3. When you agree with a post, how likely are you to retweet the post to other WeChat friends?	1.0	5.0	2.7	1.1
7.4. When you agree with a post, how likely are you to just keep it to yourself?	1.0	5.0	3.2	1.0
8.1. When you disagree with a post, how likely are you to click the *like* button?	1.0	4.0	1.7	0.8
8.2. When you disagree with a post, how likely are you to make a comment?	1.0	4.0	2.2	1.1
8.3. When you disagree with a post, how likely are you to retweet the post to other WeChat friends?	1.0	4.0	1.8	0.9
8.4. When you disagree with a post, how likely are you to just keep it to yourself?	1.0	5.0	3.8	1.1
8.5. When you disagree with a post, how likely are you to block the author of the post?	1.0	5.0	2.5	1.2
9. Do you think WeChat should add a *Dislike* button?	1	5	2.3	1.1
